# Losing one’s religion: relationships between autistic and schizotypal traits, religiosity, spirituality, and faith changes

**DOI:** 10.3389/fpsyg.2026.1689818

**Published:** 2026-03-18

**Authors:** Quinn Smith, Nancy Yang, Sam M. Doesburg, Bernard Crespi

**Affiliations:** 1 Department of Psychology, Simon Fraser University, Burnaby, BC, Canada; 2 Department of Biomedical Physiology and Kinesiology, Simon Fraser University, Burnaby, BC, Canada; 3 Department of Biology, Simon Fraser University, Burnaby, BC, Canada

**Keywords:** autism, deconversion, religion, religious cognition, religious conversion, schizotypy, spiritual experiences, spirituality

## Abstract

**Objective:**

This study aimed to investigate the relationships between positive schizotypal and autistic traits and life-changing religious or spiritual experiences and faith changes, in those of relatively diverse religious affiliations.

**Methods:**

A total of 2091 participants completed virtual questionnaires measuring autistic traits, positive schizotypy, and religious or spiritual experiences and faith changes. Participants who did or did not report a life-changing religious or spiritual experience were compared on autistic and positive schizotypal trait measures and their respective subscales. Those who reported a gain in faith, a loss in faith, neither of these, or both, were also compared on the trait measures and subscales.

**Results:**

Participants who reported a life-changing religious or spiritual experience or who experienced a gain in faith were lower in autistic social traits and higher in positive schizotypy. Participants who experienced a loss in faith also showed higher positive schizotypy. Those who reported no changes in faith showed greater autistic imaginative traits.

**Conclusion:**

These results extend prior findings to a more diverse sample and faith changes, which have seldom been previously studied in relation to autistic and schizotypal traits. Potential explanations of results provide testable hypotheses for future study, focusing on mentalizing for autistic traits, and perceptual volatility and group member distrust for positive schizotypy.

## 1 Introduction

Religious or spiritual experiences and faith changes are among the most influential events in religious history. Despite the historical and present importance of these aspects of religiosity and spirituality (R/S), their social and political weight has made empirical study difficult. Religious conversion is on the extreme side of gains in faith, where a person adopts a new religious affiliation and identity (Leuba, 1896). Losses in faith are similar, with the most extreme change being deconversion or disaffiliation—renouncing one’s faith and leaving a religious affiliation and identity. Studying faith changes, especially faith loss, is of particular interest due to current trends in religious affiliation in Western countries. In Canada, the proportion of individuals identifying as Christian is decreasing, while those identifying with non-Christian religious groups and those not identifying with any religion are increasing (Reimer and Hiemstra, 2018). Although this trend, especially the increase in non-Christian religious affiliations, may be driven at least somewhat by demographic changes, it could also be a result of individual-level shifts in faith. Particularly among younger generations, being a religious “none” (describing oneself as not having a religion) is becoming increasingly common, though this group is heterogeneous in the beliefs that individuals may hold (Wilkins-Laflamme, 2023). Among religious nones, individuals may be secular, or they may describe themselves as “spiritual but not religious”, indicating some sense of personal spirituality without religious affiliation (Wilkins-Laflamme, 2023). Using 2011 data, Reimer and Hiemstra (2018) found that the number of individuals identifying as spiritual increased by six times compared to what it was when the same participants were children, largely due to movement from other religious groups, and especially Christianity, to the “Spiritual” group. Searching for potential reasons for faith changes or personal traits related to faith changes is a natural direction of inquiry given current trends.

A variety of personality, social, cultural, and institutional factors have been identified to influence changes in faith (Gooren, 2007). When Jewish, Catholic, Bahai, and Hare Krishna converts and long-term members were compared to one another, converts showed more rigid current beliefs, negative perceptions of their parents, absent fathers, childhood and adolescent trauma, and personal stress than long-term members of the same faith groups (Ullman, 1982). In Norwegian Christian converts, life events, spiritual experiences, community interactions, interest in personal development, and finding life meaning were identified as contributors to increased faith (Nygaard et al., 2022). In another study, both Christian converts and deconverts showed some small differences in personality features such as agreeableness, specifically at the time of faith change (Bleidorn et al., 2024). Attachment style also appears to be related to faith changes, with insecure-anxious and insecure-avoidant attachment styles being related to both conversion and deconversion, while secure attachments were related to persistence within the same faith (Paloutzian et al., 1999; Zarzycka et al., 2024). Longitudinal studies have found support for the hypothesis that personality and other factors differ between converts or deconverts before faith change occurs, rather than personality change arising as a result of faith change (Hui et al., 2018; Paloutzian et al., 1999). Still, different definitions of what conversion means and a great variety in the time course and preceding circumstances of faith change complicate the integration of study findings. Further research is needed to more fully understand the role of religious context, emotions, personal characteristics, and identity factors that relate to changes in faith (Snook et al., 2019). Other individual traits that have been studied in relation to R/S, such as positive schizotypy and autistic traits, also provide opportunities to better understand cognitive factors related to faith changes.

Positive schizotypy is of interest for its relationship to R/S due to the overlap between delusional and religious beliefs (Peters, 2001). Positive schizotypy describes a set of traits similar to positive schizophrenia symptoms but with a lesser impact on day-to-day functioning, including odd beliefs or magical thinking, unusual perceptual experiences, suspiciousness, and ideas of reference (American Psychiatric Association, 2022). These traits are present in the general population and are elevated in groups of people with fringe religious or paranormal beliefs (Peters, 2001). Several studies observed relationships between non-clinical cognitive-perceptual/positive schizotypal traits and increased R/S (Breslin and Lewis, 2015, 2017; Hancock, 2011; Maltby and Day, 2002; Unterrainer and Lewis, 2014; Wlodarski and Pearce, 2016). In particular, magical thinking or ideation (a tendency to believe that seemingly separate events are related and influenced by ones actions, thoughts, or the supernatural) has been related to greater religious preoccupation (Diduca and Joseph, 1997), and has been observed to predict prayer frequency, type, and positive prayer experiences (Breslin and Lewis, 2015, 2017). Unusual perceptions, such as slight morphing of faces or hearing thoughts out loud, were also found to be related to greater religiosity, but only in men (White et al., 1995). Positive schizotypy as a whole is related to people identifying as spiritual but not religious (Willard and Norenzayan, 2017), which suggests a possible distinction between schizotypy’s relationship with religiosity versus spirituality.

Differences in mentalizing (the ability to infer mental states of others; Hutsebaut et al., 2023), agency attribution, and belief stability may explain the relationship between positive schizotypy and R/S and could also extend this relationship to faith changes. Higher willingness to ascribe free will, emotions, intentions, and consciousness (agency) to non-human objects and entities was found to be related to positive schizotypy in prior research (Wlodarski and Pearce, 2016). There was also a greater propensity to perceive intentionality in randomness (apophenia) seen in conjunction with greater positive schizotypy (Fyfe et al., 2008). Positive schizotypy is additionally linked to openness, especially maladaptively high openness or experiential permeability, a loosening of boundaries between one’s inner and outer world (Piedmont et al., 2012). This can result in “loose” cognition, unstable beliefs, and a fluctuating sense of identity. Together, unstable beliefs and increased perception of intentionality or meaning could lead to individuals higher in positive schizotypy being more likely to experience changes in R/S beliefs over time. Given the instability in identity as well, this could look like leaving and joining different religious groups.

Autistic traits, including difficulties with social skills and attention switching, more rigid and less social imagination, preference for routine, and fascination with numbers or patterns, are seen in non-clinical populations and thought to exist on a continuum with clinical autism as an extreme expression of these traits (Constantino and Todd, 2003; Ruzich et al., 2015). A number of studies have provided evidence that higher autistic traits are associated with lower religiosity, spirituality, or qualitatively different experiences of God and religion (Crespi et al., 2019; Norenzayan et al., 2012; Schaap-Jonker et al., 2013), while other studies have found no relationship between autistic traits and religiosity (Eklbad and Oviedo, 2017; Reddish et al., 2016). Of commonly measured autistic traits, difficulties with social skills and more rigid and less social imagination may be most related to reduced spirituality and belief in God (Crespi et al., 2019), though evidence is relatively limited. Cognitive features of those with higher autistic traits, including heightened mechanistic cognition and systematizing, cognitive rigidity, and reduced mentalizing, make autistic traits of interest to R/S research (Burnos and Kopacz, 2025; Crespi et al., 2019). To extend this notion to faith changes, reduced mentalizing could result in those higher in autistic traits having difficulty understanding religious ideologies involving unseen forces of agency, leading to faith loss over time. Cognitive rigidity could potentially discourage individuals higher in autistic traits from changing faith groups.

While past studies have investigated varied aspects of R/S, many different R/S measures were used. This makes it difficult to synthesize findings. Furthermore, faith changes in particular have not been investigated in relation to autistic or schizotypal traits. There is also a lack of studies investigating religious groups composed of individuals other than Christians and non-religious people, making it unclear if the relationships that have been observed apply more broadly across religions. Christianity is especially different from polytheistic religions such as Hinduism, and follows different traditions and ideology than other religions, so it could be that relationships between psychological traits and R/S in these groups would differ.

The present study aimed to investigate the relationships between autistic and positive schizotypal traits and reports of R/S experiences and faith changes in a group composed of varied religious affiliations. We hypothesized that autistic traits would be lower in groups reporting R/S experiences and changes in faith, and positive schizotypal traits would be greater in groups reporting life-changing religious and spiritual experiences and faith changes.

## 2 Methods and materials

### 2.1 Participants

A total of 2091 participants were recruited through an online research participation portal hosted by Simon Fraser University. This system is used to recruit for online studies and provides users with a unique system ID to avoid duplicate responses. It primarily captures undergraduate psychology students who participate in studies for course credit in first-year classes. As such, most participants were undergraduate students taking psychology classes at Simon Fraser University, and others were mostly staff, faculty, or graduate students at Simon Fraser University. To avoid biasing data wherever possible, missing data only resulted in participants being entirely excluded if the missing data made calculating total and subscale scores not possible. For this reason, only 12 participants missing questionnaire data were removed from all analyses; 11 participants missing sex information and 13 missing age information were excluded only where sex or age was being used in a given analysis.

### 2.2 Measures

#### 2.2.1 Autistic traits

Autistic traits were measured using the abridged 28-item version of the Autism-Spectrum Quotient (AQ-Short; Hoekstra et al., 2011), a self-report measure designed for adults with normal intelligence. The AQ-Short was developed for use in studies and as a screening tool to gage whether further autism assessment was recommended. It contained five domains (example items included in brackets): Social Skills (“I find it hard to make new friends”), Routine (“New situations make me anxious”), Attention Switching (“I frequently get strongly absorbed in one thing”), Imagination (“I find it difficult to work out people’s intentions”), and Numbers/Patterns (“I notice patterns in things all the time”). Each domain was scored on a four-point Likert scale with answer options of “1 = definitely agree”; “2 = slightly agree”; “3 = slightly disagree”; and “4 = definitely disagree”. A higher score indicated greater autistic traits, including difficulties with social skills, attention switching, imagination, a preference for routine, and fascination with numbers or patterns. The AQ-Short showed good internal consistency, a continuous distribution, and high specificity and sensitivity in clinical groups and controls from the Netherlands and the UK (Hoekstra et al., 2011). In the present sample, the internal consistency of the AQ-Short was variable. The overall AQ-Short and the Social Skills subscale had Cronbach’s *α* values of 0.76 and 0.80, respectively. The Routine, Attention Switching, Imagination, and Numbers/Patterns subscales all had Cronbach’s α values between 0.48 and 0.64, with Attention Switching and Routine on the low end and Imagination and Numbers/Patterns on the higher end.

#### 2.2.2 Positive schizotypal traits

Positive schizotypal traits were measured using items from the Cognitive-Perceptual higher order subscale of the Schizotypal Personality Questionnaire–Brief Revised Updated (SPQ-BRU; Davidson et al., 2016). The SPQ-BRU was designed for and tested on non-clinical young adult samples, with higher scores indicating greater positive schizotypy. The Cognitive-Perceptual subscale of the SPQ-BRU used in this study contained 14 items, encompassing four lower-order subscales. These subscales included (example items in brackets) the following: Ideas of Reference (“I sometimes feel that other people are watching me”), Magical Thinking (“I have had experiences with astrology, seeing the future, UFOs, ESP, or a sixth sense”), Suspiciousness (“I often feel that others have it in for me”), and Unusual Perceptions (“Everyday things can seem unusually large or small”). The Cognitive-Perceptual subscale was used due to relationships with R/S being primarily relevant to positive schizotypal traits. The SPQ-BRU factors showed generally good reliability, convergent and discriminant validity, and stability in test samples (Davidson et al., 2016). In the present sample, Cronbach’s *α* was 0.81 for the whole SPQ-BRU-CogPer, and 0.56, 0.64, 0.76, and 0.73 for the subscales of Unusual Perceptions, Suspiciousness, Magical Ideation, and Ideas of Reference, respectively.

#### 2.2.3 Life-changing religious or spiritual experiences and faith changes

Life-changing religious or spiritual experiences and faith changes were identified using the Brief Multidimensional Measure of Religiousness/Spirituality (BMMRS; Fetzer Institute, 1999; Masters, 2013), a scale designed for health research that included 38 items from 11 domains, separating religiousness and spirituality. Domains included Daily Spiritual Experiences, Values/Beliefs, Forgiveness, Private Religious Practices, Religious and Spiritual Coping, Religious Support, Religious/Spiritual History, Commitment, Organizational Religiousness, Religious Preference, and Self-Ranked Religiousness and Spirituality. For the present study, only the questions from the Religious/Spiritual History section were used. Construct validity of the BMMRS has been supported (Masters et al., 2009).

The Religious/Spiritual History section consisted of three dichotomous questions. The questions asked if the individual had ever had a life-changing religious or spiritual experience, a significant gain in faith, or a significant loss in faith. Individuals who answered “yes” to having a life-changing religious or spiritual experience were placed in one group (“R/S experience” group), while individuals who answered “no” to this question created a “no R/S experience” group. For faith gains and losses, four groups were made: individuals who had experienced both a gain and a loss in faith (gain/loss), those who had only experienced a gain in faith (gain only), those who had only experienced a loss in faith (loss only), and those who had neither a gain nor a loss in faith (no changes).

### 2.3 Procedure

Data were collected virtually with no time constraints. Participants completed the AQ-Short first, then the SPQ-BRU, the BMMRS, and a demographic survey that asked about sex, age, ethnicity, sexual orientation, handedness, and self-report religious affiliation were completed.

To determine religious affiliation, participants responded to the questions, “Do you consider yourself a religious person?” and “What is your religion?” in the demographics section. The latter question had multi-select options for participants to choose from and an open response box if they wished to self-describe. The options included “Islam,” “Atheism/Agnosticism,” “Buddhism,” “Catholicism,” “Christianity,” “Hinduism,” “Jainism,” “Sikhism,” “Pantheism,” “Taoism,” “Shinto,” “Gnosticism,” “Stoicism,” “Bahai,” “Indigenous American Religions,” “Judaism,” “Zoroastrianism,” “Paganism,” and “Prefer not to say.”

For the most part, individuals were placed in groups based on the option that they selected or described themselves as, with some exceptions. Anyone who selected “Catholicism,” “Christianity,” or indicated a form of Christian faith in the open response was placed into the group labeled “Christianity,” as participants greatly varied in the degree of detail they provided regarding their specific denomination. If a participant’s response indicated spirituality outside of specific religious groups, a group of “spiritual but not religious” (SBNR) was assigned. Participants who answered “no” to being a religious person, who described themselves as not having a religion or faith, or who selected the option of “Atheism/Agnosticism” were placed in the “Atheism/Agnosticism/Non-Religious” group. Although Atheism, Agnosticism, and being non-religious do not necessarily correspond to the same set of beliefs, the choice was made to combine them for several reasons. First, this was the grouping suggested by the religion and spirituality measure used (Fetzer Institute, 1999). Second, and this applied generally throughout grouping, the groups needed to be aggregated to provide a meaningful size for interpretation and to avoid extremely fragmented groups of highly specific participant identities. Third, participants’ responses followed various patterns that made it difficult to separate these identities. For example, participants who chose the “Atheism/Agnosticism” option may have selected either “Yes” or “No” to identifying themselves as a religious person. Some who answered “No” to being a religious person proceeded to not answer the following question about affiliation. Given that the primary purpose of grouping participants by religious affiliation was simply to get an idea about the religious diversity present in the sample, it seemed more appropriate to aggregate these groups than to attempt to make sense of the various patterns or to exclude any of these data.

The BMMRS also asked about religious affiliation in the open-response question “What is your current religious affiliation?.” Answers to this question were not considered in most cases, because it was more difficult to standardize answers given in this form. However, they were used to classify participants in cases where a participant answered “Prefer not to say” or did not give an answer in the demographic questions but clearly indicated a religious preference in the BMMRS question. For participants whose response was unsure, conflicting, unclear regarding religious affiliation, or if participants reported having multiple distinct religious affiliations, a group of “NA” was given. Again, this was to avoid having groupings that were too fragmented to meaningfully interpret.

### 2.4 Statistical analysis

#### 2.4.1 Data cleaning, significance, and error correction

Based on skewness, kurtosis, and visual assessment of distributions of participant scores, data from the AQ-Short, SPQ-BRU, and BMMRS were found to be normally distributed. Likert-type items were treated as interval data in parametric analyses given the large sample size, normality of scale score distributions, use of scale or subscale scores rather than individual item scores, and that t- and F-tests tend to be robust to violations of interval data assumptions (Carifio and Perla, 2008; Carifio and Perla, 2007; Heeren and D’Agostino, 1987; Norman, 2010). False discovery rate (FDR) correction using the Hochberg method was used to correct for multiple comparisons where a set of tests was being performed; 95% confidence intervals for post-hoc pairwise comparisons were adjusted with Bonferroni correction.

#### 2.4.2 Life-changing religious or spiritual experiences and autistic and schizotypal traits

Independent samples t-tests were used to investigate whether the “R/S experience” and “no R/S experience” groups differed on the AQ-Short and SPQ-BRU totals or subscales. Welch’s correction was applied to account for inflation of error rates.

#### 2.4.3 Faith changes and autistic and schizotypal traits

A one-way between-subjects ANOVA was used to investigate how faith change groups differed in the AQ-Short and SPQ-BRU-CogPer totals and subscales. Effect sizes of ANOVA models were reported with *η*^2^. Type III sums of squares decomposition was used in ANOVA models to account for differing group sizes. A significant ANOVA was followed up with pairwise t-tests, and the effect size was reported as Cohen’s *d*. Test assumptions were checked statistically and/or visually.

#### 2.4.4 Ethics and availability

Ethics approval was granted by the Simon Fraser University Ethics Board before and during data collection (REB#30001391, titled “Autism and Positive Schizotypy as Windows into Religious Neurocognition”). Data, study materials, and analysis code will be shared upon request to the corresponding author by email.

## 3 Results

### 3.1 Sample

The descriptive statistics for all individuals in the study and in each of the analyzed groups is presented in Table 1. The sample was somewhat heterogeneous in religious affiliation, with 614 Atheists/Agnostics/Non-religious (30%), 606 Christians (29%), 311 Sikhs (15%), 122 Muslims (6%), 92 Buddhists (4%), 71 Hindus (3%), 72 (3%) endorsing one of 12 religious affiliations outside of these larger groups, and 191 (9%) having a religious affiliation that was not reported, a combination of different distinct religious affiliations, or unable to be determined due to conflicting or uncertain responses. The sample contained 1,448 women (70%), 594 men (29%), 6 participants reporting a sex outside of male or female (0.3%), and 20 participants who preferred not to report sex (1%). The groups analyzed varied in size, reflecting overall lower numbers of participants who reported life-changing religious or spiritual experiences and faith changes than those who did not. A total of 514 participants answered “yes” to having a life-changing religious or spiritual experience, and 1,565 answered “no.” For faith changes, 800 participants reported no changes in faith, 404 reported only faith loss, 474 reported only faith gain, and 401 reported both gain and loss. All groups had a mean age of 20 years. Based on mean values alone, the groups appeared generally similar in full-scale and subscale scores for the AQ-Short and SPQ-BRU-CogPer.

**Table 1:** Descriptive statistics of the whole sample and the analyzed subgroups.

Variable	Full sample	Gain/loss	Gain only	Loss only	No changes	No R/S experience	R/S experience
	*N* = 2079	*N* = 401	*N* = 474	*N* = 404	*N* = 800	*N* = 1,565	*N* = 514
Age (years)	20 ± 3	20 ± 3	20 ± 3	20 ± 3	20 ± 2	20 ± 2	20 ± 3
AQ-short total	65 ± 9	65 ± 9	65 ± 8	66 ± 10	65 ± 9	66 ± 9	64 ± 8
Social skills	19 ± 5	19 ± 5	19 ± 4	19 ± 5	19 ± 5	19 ± 5	18 ± 4
Routine	11 ± 2	11 ± 2	11 ± 2	11 ± 2	11 ± 2	11 ± 2	10 ± 2
Attention switching	10 ± 2	10 ± 2	10 ± 2	11 ± 2	11 ± 2	10 ± 2	10 ± 2
Imagination	16 ± 4	16 ± 4	16 ± 4	16 ± 4	16 ± 4	16 ± 4	16 ± 4
Numbers/patterns	12 ± 3	12 ± 3	12 ± 3	12 ± 3	12 ± 3	12 ± 3	13 ± 3
SPQ-BRU-CogPer	41 ± 9	43 ± 9	40 ± 9	43 ± 9	39 ± 9	40 ± 9	42 ± 9
Unusual perceptions	11 ± 3	12 ± 3	11 ± 3	12 ± 3	11 ± 3	11 ± 3	12 ± 3
Ideas of reference	10 ± 3	11 ± 3	10 ± 3	11 ± 3	10 ± 3	10 ± 3	10 ± 3
Suspiciousness	9 ± 3	10 ± 3	9 ± 3	10 ± 3	9 ± 3	9 ± 3	10 ± 3
Magical ideation	10 ± 4	10 ± 4	10 ± 4	10 ± 4	9 ± 4	9 ± 4	10 ± 4

Values are presented as mean ± standard deviation. Subscales of the AQ-Short and SPQ-BRU-CogPer are shown in the rows beneath the total score.

### 3.2 Differences in autistic or positive schizotypal traits based on life-changing religious or spiritual experiences

**Figure 1:**
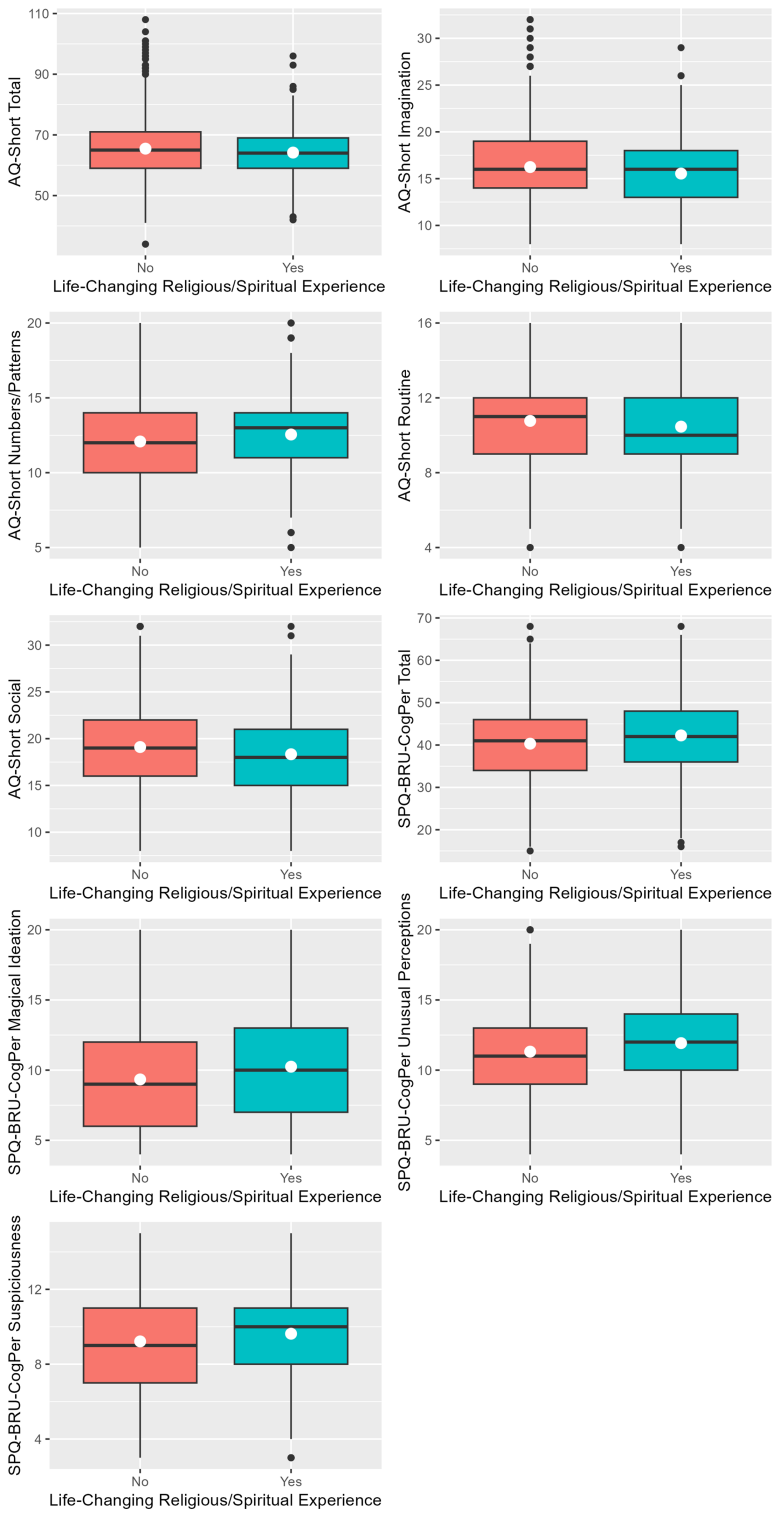
Autistic trait and positive schizotypy subscale scores were compared between participants who did or did not report a life-changing religious or spiritual experience. Means are indicated by white circles. Black lines represent medians. Only comparisons that were found to be significant with FDR correction are displayed.

Differences were identified between participants who did or did not report a life-changing religious or spiritual experience in their autistic and positive schizotypal traits (*p* < 0.01). These results are also shown in Table 2. Those who reported a life-changing religious or spiritual experience were higher in most positive schizotypal traits, including the SPQ-BRU-CogPer total (95% CI [−2.82, −1.07], *d* = −0.22), Unusual Perceptions (95% CI [−0.92, −0.29], *d* = −0.19), Suspiciousness (95% CI [−0.67, −0.15], *d* = −0.16), and Magical Ideation (95% CI [−1.29, −0.52], *d* = −0.24). This group also scored higher than the “no R/S experience” group on the AQ-Short Numbers/Patterns (95% CI [−0.75, −0.18], *d* = −0.16). The “no R/S experience” group scored higher on the other AQ-Short variables, including the total (95% CI [0.45, 2.14], *d* = 0.15), Social Skills (95% CI [0.33, 1.21], *d* = 0.17), Routine (95% CI [0.09, 0.52], *d* = 0.01), and Imagination (95% CI [0.33, 1.05], *d* = 0.19). Only the Attention Switching subscale of the AQ-Short and the Ideas of Reference subscale of the SPQ-BRU-CogPer showed no differences between groups. Figure 1 contains boxplots displaying score distributions and central tendency measures of the AQ-Short and SPQ-BRU-CogPer between those who did and did not report a life-changing R/S experience to provide a visual representation of these findings. Multiple comparison adjustment was based on the 11 tests performed.

**Table 2:** Results of independent samples t-tests comparing AQ-short and SPQ-BRU-CogPer scores between those who did and did not report a life-changing religious or spiritual experience.

Variable	*t(df)*	*p*	95% CI	Cohen’s *d*
AQ-short total	**3.02 (992)**	**0.0026**	**0.45, 2.14**	**0.15**
Social skills	**3.46 (948)**	**0.0006**	**0.33, 1.21**	**0.17**
Routine	**2.80 (904)**	**0.0053**	**0.09, 0.52**	**0.01**
Attention switching	1.36 (845)	0.1757	−0.07, 0.38	-
Imagination	**3.79 (932)**	**0.0002**	**0.33, 1.05**	**0.19**
Numbers/patterns	**−3.20 (881)**	**0.0014**	**−0.75, −0.18**	**−0.16**
SPQ-BRU-CogPer total	**−4.38 (876)**	**<0.0001**	**−2.82, −1.07**	**−0.22**
Unusual perceptions	**−3.78 (872)**	**0.0002**	**−0.92, −0.29**	**−0.19**
Ideas of reference	−0.18 (884)	0.8539	−0.29, 0.24	-
Suspiciousness	**−3.14 (884)**	**0.0017**	**−0.67, −0.15**	**−0.16**
Magical ideation	**−4.65 (863)**	**<0.0001**	**−1.29, −0.52**	**−0.24**

Degrees of freedom were adjusted using Welch’s correction to account for differences in group variances. *df*, degrees of freedom, CI, confidence interval of mean difference. Subscales of the AQ-Short and SPQ-BRU-CogPer are listed below the rows for the total scale scores. *p*-values are unadjusted. Results that were significant after FDR correction are bolded.

### 3.3 Differences in autistic or positive schizotypal traits based on faith changes

**Figure 2:**
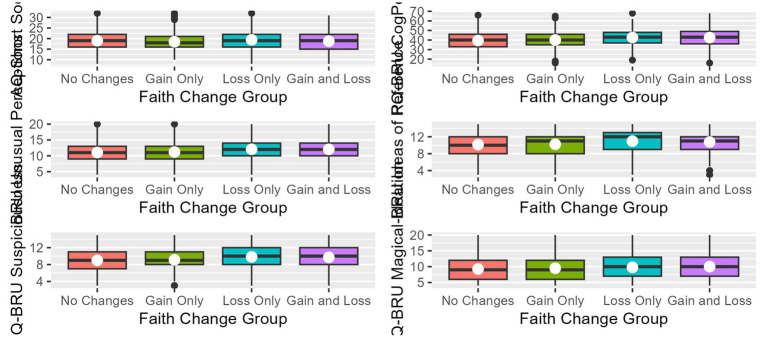
Boxplots showing mean scores of autistic traits and positive schizotypy among faith change groups. White dots represent means and black lines represent medians. Only AQ-Short and SPQ-BRU-CogPer subscales and totals that had significant ANOVA results are shown.

ANOVA tables examining the differences in AQ-Short or SPQ-BRU-CogPer among faith change groups are reported in Table 3, and results from subsequent pairwise-comparison analyses are shown in Table 4. Figure 2 contains boxplots displaying score distributions and central tendency measures on the AQ-Short and SPQ-BRU-CogPer between faith change groups. Including religious affiliation in ANOVA models did not indicate an effect of religious affiliation on the results. These results will additionally be described.

**Table 3:** Results of one-way between-subjects ANOVA comparing AQ-short and SPQ-BRU-CogPer scores between faith change groups.

Variable	*F(3,2075)*	*p*	*η^2^*
AQ-short total	2.31	0.0743	-
Social skills	**3.02**	**0.0287**	**0.0003**
Routine	2.38	0.0679	-
Attention switching	2.45	0.0617	-
Imagination	**3.92**	**0.0084**	**0.0003**
Numbers/patterns	2.33	0.0728	-
SPQ-BRU-CogPer total	**18.63**	**<0.0001**	**0.0012**
Unusual perceptions	**15.46**	**<0.0001**	**0.0016**
Ideas of reference	**11.36**	**<0.0001**	**0.0010**
Suspiciousness	**12.96**	**<0.0001**	**0.0014**
Magical ideation	**4.02**	**0.0073**	**0.0008**

Significant uncorrected *p*-values after FDR correction are bolded. Subscales of the AQ-Short and SPQ-BRU-CogPer appear after the total score rows.

**Table 4:** Results of pairwise comparisons between faith change groups on AQ-short and SPQ-BRU-CogPer scores.

Variable	Groups compared	*p*	95% CI	Cohen’s *d*
AQ-short imagination	No changes–gain only	0.0838	−0.20, 0.95	-
	No changes–loss only	0.0475	−0.15, 1.06	-
	Gain only–loss only	0.759	−0.59, 0.75	-
	**No changes–gain/loss**	**0.0011**	**0.15, 1.36**	**0.20**
	Gain only–gain/loss	0.14	−0.30, 1.05	-
	Loss only–gain/loss	0.26	−0.40, 0.99	-
AQ-short social skills	No changes–gain only	0.0836	−0.24, 1.16	-
	No changes–loss only	0.114	−1.18, 0.30	-
	**Gain only–loss only**	**0.0037**	**−1.72, −0.08**	**−0.20**
	No changes–gain/loss	0.445	−0.52, 0.96	-
	Gain only–gain/loss	0.43	−1.07, 0.58	-
	Loss only–gain/loss	0.0421	−0.20, 1.51	-
SPQ-BRU-CogPerTotal	No changes–gain only	0.236	−1.93, 0.74	-
	**No changes–loss only**	**<0.0001**	**−4.56, −1.75**	**−0.36**
	**No changes–gain/loss**	**<0.0001**	**−4.48, −1.67**	**−0.35**
	**Gain only–loss only**	**<0.0001**	**−4.11, −1.00**	**−0.30**
	**Gain only–gain/loss**	**<0.0001**	**−4.03, −0.92**	**−0.29**
	Loss only–gain/loss	0.89	−1.53, 1.71	-
Unusual perceptions	No changes–gain only	0.446	−0.62, 0.34	-
	**No changes–loss only**	**<0.0001**	**−1.52, −0.51**	**−0.32**
	**No changes–gain/loss**	**<0.0001**	**−1.50, −0.49**	**−0.32**
	**Gain only–loss only**	**<0.0001**	**−1.43, −0.32**	**−0.28**
	**Gain only–gain/loss**	**<0.0001**	**−1.42, −0.29**	**−0.27**
	Loss only–gain/loss	0.912	−0.56, 0.61	-
Ideas of reference	No changes–gain only	0.638	−0.48, 0.33	-
	**No changes–loss only**	**<0.0001**	**−1.23, −0.38**	**−0.30**
	**No changes–gain/loss**	**0.0002**	**−1.03, −0.18**	**−0.23**
	**Gain only–loss only**	**<0.0001**	**−1.21, −0.26**	**−0.28**
	**Gain only–gain/loss**	**0.0030**	**−1.01, −0.06**	**−0.21**
	Loss only–gain/loss	0.278	−0.29, 0.69	-
Suspiciousness	No changes–gain only	0.382	−0.53, 0.26	-
	**No changes–loss only**	**<0.0001**	**−1.22, −0.39**	**−0.31**
	**No changes–gain/loss**	**<0.0001**	**−1.14, −0.30**	**−0.28**
	**Gain only–loss only**	**0.0001**	**−1.14, −0.21**	**−0.27**
	**Gain only–gain/loss**	**0.0008**	**−1.05, −0.13**	**−0.23**
	Loss only–gain/loss	0.635	−0.39, 0.57	-
Magical ideation	No changes–gain only	0.247	−0.84, 0.33	-
	No changes–loss only	0.0242	−1.14, 0.09	-
	**No changes–gain/loss**	**0.0013**	**−1.37, −0.14**	**−0.20**
	Gain only–loss only	0.297	−0.95, 0.41	-
	Gain only–gain/loss	0.0551	−1.18, 0.19	-
	Loss only–gain/loss	0.398	−0.94, 0.48	-

Groups whose *p*-values were significant after FDR correction are shown in bold. CI, confidence interval, adjusted with the Bonferroni method for six comparisons.

For the AQ-Short, only the Imagination (*p* = 0.0084, *η^2^* = 0.0003) and Social Skills (*p* = 0.0287, *η^2^* = 0.0003) subscales differed between faith change groups. Those who reported no changes in faith scored higher on the Imagination subscale (indicating reduced social imagination) than did those who reported both a gain and a loss in faith (M = 16, SD = 4, *p* = 0.0011, 95% CI [0.15, 1.36], *d* = 0.20). The group that reported only a loss in faith scored higher on the Social Skills subscale (indicating social difficulties) than the gain only group (M = 19, SD = 5 and 4, *p* = 0.0037, 95% CI [−1.72, −0.08], *d* = −0.20). This reflects greater autistic social traits (preference for solitude, difficulty in social situations) in those who experienced a loss in faith compared to those who experienced a gain in faith. The Routine, Attention Switching, and Numbers/Patterns subscales did not produce significant results.

The total SPQ-BRU-CogPer (*p* < 0.0001, *η^2^* = 0.0012), and each of its subscales (*p* < 0.0001 for all but Magical Ideation, which was *p* = 0.0073), showed significant differences between faith change groups. For the Total, Unusual Perceptions, Ideas of Reference, and Suspiciousness, both the gain/loss (Total: M = 43, SD = 9, Unusual Perceptions: M = 12, SD = 3, Ideas of Reference: M = 11, SD = 3, Suspiciousness: M = 10, SD = 3) and loss only (Total: M = 43, SD = 9, Unusual Perceptions: M = 12, SD = 3, Ideas of Reference: M = 11, SD = 3, Suspiciousness: M = 10, SD = 3) groups scored higher than each of the no change (Total: M = 39, SD = 9, Unusual Perceptions: M = 11, SD = 3, Ideas of Reference: M = 10, SD = 3, Suspiciousness: M = 9, SD = 3) and gain only (Total: M = 40, SD = 9, Unusual Perceptions: M = 11, SD = 3, Ideas of Reference: M = 10, SD = 3, Suspiciousness: M = 9, SD = 3) groups. Put another way, any group that reported a loss in faith indicated more of these schizotypal traits than any group not reporting a loss in faith. The gain/loss and loss only groups did not differ from one another, nor did the no change and gain only groups. Magical Ideation scores were only higher in the gain/loss group compared to the no change group (M = 10 and 9, SD = 4, *p* = 0.0013, 95% CI [−1.37, −0.14], *d* = −0.20). All effect sizes were small, with Cohen’s *d* values between −0.20 and −0.36. In summary, groups that reported a loss in faith (alongside a gain or not) tended to score higher on measures of positive schizotypy than groups who reported no losses in faith (no changes at all or only gains in faith).

For all ANOVA models, multiple comparison correction was applied initially based on the 11 F-tests, and then for each batch of six comparisons between faith change groups. It is important to emphasize that, because of the large sample size, small *p*-values may have been obtained despite small or very small effect sizes. Because of this, results should be interpreted with caution.

## 4 Discussion

### 4.1 Life-changing religious and spiritual experiences

#### 4.1.1 Summary

Our study found that those who reported having had a life-changing religious or spiritual experience scored higher in SPQ-BRU-CogPer total, Unusual Perceptions, Suspiciousness, Magical Thinking, and AQ-Short Numbers/Patterns than those who did not. These results mirror prior findings in which those higher in positive schizotypy were found to report more spiritual experiences (Breslin and Lewis, 2017; Maltby and Day, 2002). Furthermore, the sample of the present study had more heterogeneity in religious affiliation compared to past work. The lack of differences between religious groups suggested that these relationships may extend beyond Atheist, Agnostic, and Christian-only groups. This would be an interesting avenue for further investigation with samples of different cultural makeups and religious backgrounds.

#### 4.1.2 Positive schizotypy and R/S experiences

Positive schizotypy has been related to greater mentalizing, increased global relative to local processing, lower scientific reasoning, and more weighting to currently held beliefs (rather than new sensory information) to make predictions (Georgiou et al., 2022; Kemp et al., 2020; Russell-Smith et al., 2010; Tarasi et al., 2023; Wlodarski and Pearce, 2016). Combining these cognitive differences with potentially increased R/S may result in those higher in positive schizotypy interpreting sensory information using preexisting spiritual or religious beliefs and ascribing agency and meaning to this information. This could happen in scenarios where those without the pre-existing beliefs and schizotypal traits would simply perceive a random event.

Investigating the timeline of R/S experiences and religious belief development would build upon this theory, as the present data cannot delineate whether R/S beliefs are present before or come to fruition following R/S experiences. Historical religious or spiritual experiences, such as those of St. Paul and St. Augustine, are often described as occurring before religious belief as inciting factors in these individuals’ religious commitment (Paloutzian et al., 1999). In the future, identifying the R/S beliefs one holds before and after these experiences is an avenue for investigation that would provide modern evidence and context to historical religious accounts of religious and spiritual experiences.

#### 4.1.3 Autistic traits and R/S experiences

The group not reporting life-changing R/S experiences had higher scores on AQ-Short Total, Social Skills, Imagination, and Preference for Routine. These traits may result in individuals having more realism-grounded, sensory-driven interpretations of their world and being less likely to ascribe agency to unseen forces such as those present in religious or spiritual experiences. Future studies should look at the role of R/S beliefs or religious affiliation in this area to determine whether lesser R/S beliefs in those higher in autistic traits result in fewer R/S experiences. They may also investigate if a lack of R/S experience due to cognitive differences is a source of reduced R/S.

### 4.2 Positive schizotypy and faith changes—faith lability

#### 4.2.1 Summary

Groups that experienced both a gain and/or a loss in faith scored higher on Total SPQ-BRU-CogPer, Unusual Perceptions, Ideas of Reference, and Suspiciousness than groups that experienced no changes or only gains in faith. Only those who experienced both a gain and a loss in faith scored significantly higher on Magical Ideation. Religious deconverts tended to show greater Openness to Experience and were more likely to identify as spiritual but not religious in prior research (Streib, 2021). Both Openness to Experience and being spiritual but not religious were also related to greater positive schizotypy (Kemp et al., 2020; Willard and Norenzayan, 2017). Furthermore, religious deconversion is more likely to be associated with avoidant or anxious attachment styles compared to secure attachment styles (Paloutzian et al., 1999; Zarzycka et al., 2024). Given that insecure attachments have been linked to positive schizotypy (Sheinbaum et al., 2013; Tiliopoulos and Goodall, 2009), the volatile faith pattern observed here may correspond to an underlying insecure interpersonal attachment style. In other words, positive schizotypy appears to be related to faith lability, with individuals higher in positive schizotypy being more likely to change R/S beliefs, perhaps multiple times, throughout their lives. This could potentially be explained by a connection between positive schizotypal traits and the combination of belief instability and group distrust.

Unusual perceptions, such as alterations to object size and faces, could reflect that one’s perception of the world is more variable. This has similarities to observed relationships between minimal self-disorders and the schizophrenia spectrum. In minimal self-disorders, individuals who later develop schizophrenia exhibit instability in their first-person perspective of the world, resulting in aberrant subjective experiences (Nelson et al., 2014). If perceptions are variable, an individual may be led toward different belief systems over time, their physical world being interpreted differently depending on their thoughts and perceptions. Alternatively, it may be the case that both perceptual and belief instability result from an underlying labile nature of representations in those who exhibit greater positive schizotypy. Magical ideation, such as belief in fortune telling or the existence of psychic forces, could result in one’s faith being impacted by randomly occurring chance events that are viewed as having a message or meaning, which could destabilize current beliefs. Putting these together, when interpretations of one’s external world are more erratic and rely on meaning derived from random events, beliefs may be less stable over time with new incoming sensory experiences.

Other aspects of positive schizotypy, such as suspiciousness and general distrust, could support faith lability differently. Being suspicious of others’ intentions could lead an individual to distrust the members of a faith group, resulting in movement from one faith group to another. Similar to suspiciousness, ideas of reference could also add to tensions with others, such as believing that innocuous conversations are conspiracies against oneself. Increased feelings of rejection and assumptions of being rejected in social settings are related to increased positive schizotypy (Kwapil et al., 2012; Premkumar et al., 2014), which supports the notion that these individuals may struggle socially in groups. Distrust of one’s in-group, rather than fluctuations in beliefs, could lead to the leaving and joining of multiple faith groups over time in these instances.

#### 4.2.2 Future directions

In future studies, identifying individuals high in positive schizotypy and following these individuals and their beliefs over time would provide valuable insight into potential faith transitions. To differentiate between changes in faith due to changes in beliefs or due to distrust in other group members, these individuals could be asked about their reasons for changes in faith. Future studies should also be specific with the wording of questions regarding changes in faith to clearly distinguish a change in beliefs from a change in religious affiliation, as this was unclear in the present study. The meaning of “loss in faith” is somewhat ambiguous and does not distinguish between those who remain in a faith group but have less involvement and those who completely disaffiliate. This wording also does not differentiate between leaving a religious group or losing spiritual or religious beliefs. Investigating faith changes with clearer questions that separate these different trajectories would specify exactly which aspects of faith change relate to positive schizotypy.

### 4.3 Autistic traits and faith changes—mentalizing and agency attribution

#### 4.3.1 Summary

AQ-Short scores for Imagination and Social Skills differed between faith change groups. The group that reported no faith changes scored higher on AQ-Short Imagination than the group that experienced both gains and losses in faith, and the group that experienced only losses in faith scored higher on AQ-Short Social Skills than the group that experienced only faith gains. Despite the name of the subscale being “Imagination,” five out of eight questions on this subscale of the AQ-Short ask about pretending and working out the internal worlds and intentions of others (Hoekstra et al., 2011), which appear to reflect mentalizing, an aspect of social cognition (Hutsebaut et al., 2023). Based on this, mentalizing and its behavioral manifestations may be key factors underpinning the faith experience of individuals high in autistic traits, leading these individuals to be less religious or spiritual. The AQ-Short defines autistic social traits as not being drawn to other people and preferring oneself or objects as company. Being part of a faith group often involves large gatherings, many new people, and doing activities with others, which may be challenging for individuals higher in autistic traits who struggle with sensory overload and navigating social cues (Hodis et al., 2025). Thus, the social behavior of those higher in autistic traits may lead to decreased participation in organized religion and a tendency to leave rather than join faith groups. Furthermore, lower mentalizing may also lead to lower R/S beliefs due to difficulty relating to and conceptualizing the internal worlds of other beings. R/S beliefs frequently rely on the ascription of agency and intentionality to unseen beings, particularly social agents.

#### 4.3.2 Numbers and patterns subscale behavior

The behavior of the AQ-Short Numbers and Patterns subscale was unexpected, as relationships followed the pattern of schizotypal traits rather than other autistic traits. This may stem from the questions on this subscale being interpreted differently than they were intended to be. Questions from the Numbers and Patterns subscale of the AQ-Short ask about “fascination” with dates and numbers and noticing patterns in one’s environment (Hoekstra et al., 2011). These may be interpreted similarly to magical thinking, with the “fascination” conveying that an individual attributes a purpose or meaning to random events or patterns. Due to its opposite behavior to the other AQ-Short subscales and alignment with measures of positive schizotypy, the validity of this subscale may need to be revisited.

### 4.4 Implications for diametric models of autistic and schizotypal traits

Diametric models of autistic and positive schizotypal traits propose that these groups of traits represent two extremes of social cognition (Crespi and Badcock, 2008). Under this model, autistic traits demonstrate relative underdevelopment of social cognition, while positive schizotypy is relative overdevelopment. The findings of the present study are a novel extension of the diametric model and research that has previously investigated the diametric model in relation to differences in religiosity and spirituality (Crespi et al., 2019). Specifically, faith changes may be another factor upon which autistic and schizotypal traits diverge, driven by previously identified divergence in aspects of cognition such as mentalizing, agency attribution, analytical skills, and belief formation (Crespi and Badcock, 2008; Georgiou et al., 2022; Norenzayan et al., 2012; Wlodarski and Pearce, 2016).

### 4.5 Limitations

Our study has several limitations. First, while the sample was relatively diverse in religious affiliation, it still had large numbers of Christians and Atheists relative to other religious groups. This made religious group comparisons underpowered. The participants were also primarily undergraduate psychology students, who are primarily young adults between the ages of 18 and 29 and hold an undergraduate education, making the results not easily extended to the general public.

Second, the results may have been influenced by lower overall religiosity and spirituality in the sample, since younger people tend to be less religious than older people (Pew Research Center, 2018). Given that faith changes tend to occur between the ages of 15 and 29 (Pew Research Center, 2022), the portion of individuals who reported no changes in faith may have been smaller had the sample been older. Future studies should incorporate samples outside of university student populations with wider age ranges, diversity in socioeconomic groups, and from different parts of the world. This would allow the theories presented here to be tested and extended to groups with greater religiosity, more time to experience changes in faith, and of different cultural demographics, and would hopefully help with any power issues that may have arisen from an imbalance in group sizes in the present study.

Third, a similar study in another cultural context would allow the cross-cultural implications of our findings to be better understood. The personal impact of changing faith, especially losing faith, may be very different in other cultures where religion is a more important aspect of daily life, and this could alter the relationships between faith changes and individual traits.

Fourth, due to the cross-sectional nature of this study, it cannot be determined if autistic and schizotypal traits are stable throughout life and thus drive religiosity and spirituality, or if these traits change with faith changes or because of religious and spiritual experiences. A longitudinal study following individuals who underwent religious conversion or deconversion saw that these people largely changed in religious practices rather than personality when they converted or deconverted (Bleidorn et al., 2024), and other longitudinal studies also saw that trait differences were present before changes in faith for converts and deconverts (Hui et al., 2017, 2018). Furthermore, beliefs in this study were treated as relatively stable characteristics, with changes in religiousness or spirituality assumed to be significant and memorable. There is evidence that beliefs may be changed more often and based on situational factors (Sharot et al., 2023). It seemed reasonable to treat religious and spiritual beliefs as relatively stable here, as situational factors during the short period of data collection were unlikely to drastically change one’s beliefs to the degree that it would impact the measures used. Overall, it appears more likely that autistic and schizotypal traits would predict changes in religiosity and spirituality (when treated as relatively stable), but future longitudinal studies are needed.

Finally, incorporating a mentalizing measure into a study similar to this one would allow for a potential mediating effect of mentalizing to be investigated in relation to autistic social and imaginative traits and faith changes or other aspects of R/S.

## 5 Conclusion

We found that those who reported a life-changing religious or spiritual experience or a loss in faith showed greater positive schizotypy than those who did not. We also saw that those who did not report a life-changing religious or spiritual experience exhibited greater autistic traits in general and in social skills, imagination, and preference for routine. While autistic and positive schizotypal traits have been studied in relation to religiosity and spirituality, exploring faith changes is a novel and relevant focus given shifting faith demographics in Western countries such as Canada. These findings add to the literature that contrasts cognitive features between positive schizotypy and autistic traits, including diametric models of autism and positive schizotypy. We suggest that positive schizotypy is related to faith lability, with unstable perception of one’s world contributing to an increased incidence of religious or spiritual experiences and more changes in beliefs over time. We also postulate that those higher in positive schizotypy may be more likely to leave faith groups due to mistrust of other group members. For autistic traits, we theorize that they primarily interact with religiosity and spirituality through lesser mentalizing, resulting in a decreased likelihood of believing in supernatural forces and leading to social interaction preferences that discourage involvement in faith groups.

Finally, our findings support and extend upon prior literature in this area by incorporating faith changes as tested features of religiosity and spirituality, providing testable hypotheses for future investigations, and acting as a novel extension to diametric models of autistic and positive schizotypal traits. The findings help illuminate potential cognitive factors underlying religious and spiritual experiences and changes in faith, some of the most spectacular cognitive-affective events in the human experience.

## Data Availability

The raw data supporting the conclusions of this article will be made available by the authors, without undue reservation.
